# c-Myc Is Essential to Prevent Endothelial Pro-Inflammatory Senescent Phenotype

**DOI:** 10.1371/journal.pone.0073146

**Published:** 2013-09-06

**Authors:** Victoria Florea, Nithya Bhagavatula, Gordana Simovic, Francisco Y. Macedo, Ricardo A. Fock, Claudia O. Rodrigues

**Affiliations:** 1 Interdisciplinary Stem Cell Institute, University of Miami Leonard M. Miller School of Medicine, Miami, Florida, United States of America; 2 Department of Molecular and Cellular Pharmacology, University of Miami Leonard M. Miller School of Medicine, Miami, Florida, United States of America; Medical University Innsbruck, Austria

## Abstract

The proto-oncogene c-Myc is vital for vascular development and promotes tumor angiogenesis, but the mechanisms by which it controls blood vessel growth remain unclear. In the present work we investigated the effects of c-Myc knockdown in endothelial cell functions essential for angiogenesis to define its role in the vasculature. We provide the first evidence that reduction in c-Myc expression in endothelial cells leads to a pro-inflammatory senescent phenotype, features typically observed during vascular aging and pathologies associated with endothelial dysfunction. c-Myc knockdown in human umbilical vein endothelial cells using lentivirus expressing specific anti-c-Myc shRNA reduced proliferation and tube formation. These functional defects were associated with morphological changes, increase in senescence-associated-β-galactosidase activity, upregulation of cell cycle inhibitors and accumulation of c-Myc-deficient cells in G1-phase, indicating that c-Myc knockdown in endothelial cells induces senescence. Gene expression analysis of c-Myc-deficient endothelial cells showed that senescent phenotype was accompanied by significant upregulation of growth factors, adhesion molecules, extracellular-matrix components and remodeling proteins, and a cluster of pro-inflammatory mediators, which include Angptl4, Cxcl12, Mdk, Tgfb2 and Tnfsf15. At the peak of expression of these cytokines, transcription factors known to be involved in growth control (E2f1, Id1 and Myb) were downregulated, while those involved in inflammatory responses (RelB, Stat1, Stat2 and Stat4) were upregulated. Our results demonstrate a novel role for c-Myc in the prevention of vascular pro-inflammatory phenotype, supporting an important physiological function as a central regulator of inflammation and endothelial dysfunction.

## Introduction

The proto-oncogene c-Myc is a transcription factor well known for its role in the regulation of proliferation, growth, differentiation and survival of many cell types [1]. Gene expression profiling studies indicated that c-Myc regulates a large number of genes involved in a wide range of cellular functions [2], suggesting an important physiological role for this transcription factor [3]. Deregulated c-Myc expression has been associated with cancer and cardiovascular disorders [4], [5]. In the vascular system, the participation of c-Myc in vascular injury and atherosclerosis by promotion of smooth muscle cell proliferation is well established [6]–[9]. In the last decade, several reports, have demonstrated a requirement for c-Myc in vascular development, suggesting an important role in endothelial cell function [10]–[13]. The phenotype described upon loss of c-Myc *in vivo* supports an important physiological role in blood vessel maturation and maintenance of vascular homeostasis. However, the molecular mechanisms by which c-Myc regulates endothelial cell function remain elusive.

Endothelial cells play an essential role in maintaining vascular homeostasis by regulating immuno-inflammatory responses, coagulation, neoangiogenesis after injury and alterations in blood flow [14]. Chronic injury to the endothelium by hemodynamic stress, vasoactive challenge, hyperlipidemia or high glucose can cause cumulative damage, often related to oxidative stress that leads to disruption of endothelial function [15]. Cells respond to injury by triggering cell death or development of senescence [16]. Senescent endothelial cells retain metabolic activity, and secrete growth factors and chemokines, that stimulate other cell types. In addition, they express high levels of adhesion molecules involved in the recruitment and attachment of inflammatory cells [17]. Endothelial senescence has been implicated in endothelial dysfunction, which is characterized by phenotypic and hemodynamic changes in blood vessels that increase the risk of cardiovascular disease (CVD), such as atherosclerosis, and associated myocardial infarction and stroke [18], [19]. Therefore, better understanding of the molecular mechanisms underlying endothelial dysfunction is crucial to improve early detection and prognosis of CVD.

In the present study we show that loss of c-Myc in human endothelial cells disrupts cell growth by triggering senescence, compromising endothelial function and vascular homeostasis. This senescent phenotype was associated with induction of a pro-inflammatory response through transcriptional activation of signaling pathways that drive inflammation. Our results suggest a novel role of c-Myc in controlling vascular inflammation and present potential targets that may be used in the treatment of endothelial dysfunction.

## Materials and Methods

### Cell Lines and Culture Conditions

Human umbilical vein endothelial cells (HUVECs) and human dermal microvascular endothelial cells (HDMECs) were purchased from Lonza and maintained according to manufacturer’s instruction in endothelial growth media (EGM-2) on tissue culture plates coated with monomeric rat tail collagen type-I (BD Biosciences). For all experiments, cells were used between passages 5–8 maximum, unless otherwise stated, and maintained under 37°C/5% CO_2_ atmosphere. For replicative senescence studies, HUVECs and HDMECs were analyzed at low (Passage 6) and high (Passage 11–12) passages. For stress-induced senescence, HUVECs were grown under confluence for 1–2 days to induce quiescence, and treated with 2 ng/ml TGF-β1 in endothelial basal media supplemented with 2% fetal bovine serum for a period of 3 days. TGF-β1 was added every day during this period. Lysates were collected for analysis of senescence-associated (SA)-β-galactosidade activity, and protein and RNA expression changes.

### Production of Lentiviral Vectors

Plasmid constructs (pGIPZ vector) expressing Human c-Myc (KD) and non-silencing control (NS) shRNAs (Thermoscientific, Open Biosystems) were used to prepare lentiviral vectors for c-Myc knockdown. Viral packaging was performed by transfection of human embryonic kidney cells (HEK 293T) with lipofectamine 2000 (Invitrogen, Life Technologies) using these plasmids and two others encoding proteins required for viral packaging, pCMV-VSV-G and GAG-Pol, in DMEM media free of serum and antibiotics. Culture supernatants containing lentiviral particles were collected 24 and 48 hours after transfection, pooled and filtered through a 45µm membrane to remove cell debris. Viral particles were concentrated by precipitation with PEG-it Virus precipitation solution (System Biosciences) according to manufacturer’s instructions. Viral titer (LPS/ml) was determined by ELISA using a commercially available kit for detection of lentivirus associated protein p24 (Cell Biolabs).

### Transduction of HUVECs with Lentiviral Vectors for c-Myc Knockdown

HUVECs were cultured in endothelial basal media (Lonza) supplemented with 2% fetal bovine serum and transduced with equal amounts of lentiviral particles of NS-control and KD shRNA constructs. After overnight incubation, culture media was replaced with fresh EGM-2. Transduction efficiency was determined by observing expression of green fluorescent protein (GFP) under a fluorescence microscope. GFP-positive cells were selected 48 hours after transduction with puromycin and cells analyzed 7 days after transduction for all experiments, unless otherwise stated. c-Myc knockdown was determined at protein and RNA level by Western blot and quantitative RT-PCR, respectively, using specific antibody (Cell Signaling, clone D84C12) and Taqman probe (Applied Biosystems, Life Technologies).

### Cell Growth and Proliferation

The effect of c-Myc loss on endothelial cell proliferation was assessed in control and knockdown HUVECs 7 days after transduction using three parameters: growth curve, population doubling and DNA synthesis. For growth curve experiments, NS and KD cells were plated at an initial density of 1×10^5^ cells/60 mm collagen coated culture dish in EGM media. The number of cells was counted every day for a period of 4 days using an automated cell counter (BioRad). For determination of population doubling, cells were counted at each passage and replated during a period up to 2 weeks after transduction, approximately when knockdown cells stopped proliferating. Population doubling was estimated using pre-designed calculator software Doubling-Time available on line http://www.doubling-time.com/compute.php?lang=ensoftware. DNA synthesis was determined at different time points by incorporation of the nucleoside analogue EdU (5-ethynyl-2′-deoxyuridine), using a commercially available kit (Invitrogen, Life Technologies). Cells were harvested at different time points ranging from 2–24 hours and processed according to manufacturer’s instructions. Immunodetection and quantification of EdU-positive cells was performed by flow cytometry using the BD LSR Fortessa cell analyzer (BD Biosciences). At least 10,000 events were computed per sample.

### Tube Formation Assay

Control and knockdown HUVECs were used for tube formation assay on basement membrane matrix 7 days after transduction under starved (overnight incubation in endothelial basal media supplemented with 0.5% fetal bovine serum) or stimulated conditions (grown in complete endothelial growth media). Cells were harvested by brief trypsinization and plated on 24-well culture dish (6×10^4^ cells/well) pre-coated with 300µl of regular growth factor basement membrane matrix (Matrigel, BD Biosciences, #354234) in endothelial basal media (Lonza) supplemented with 0.1% bovine serum albumin. After a period of 5 hours, samples were analyzed for tube formation potential by quantification of the number of tubes formed and measurement of tube length using ImageJ Software. At least 6 bright field images were collected at 10×magnification from random fields for each sample from independent experiments.

### Extracellular-Matrix Adhesion Assay

Interaction of control and knockdown HUVECs with extracellular-matrix proteins was determined using a microplate colorimetric assay kit commercially available (Millipore, #ECM540). Cells were collected by brief trypsinization and equal numbers (1×10^5^ cells per well) plated and incubated for 1 hour at 37°C/5% CO_2_ atmosphere. After this time, cells were stained with crystal violet, washed 3 times and lysed. Colorimetric detection of crystal violet released into the supernatant, which is proportional to the number of attached cells, was determined using a SpectraMax M5 plate reader (Molecular Devices). Results were expressed as absorbance units.

### Quantitative Detection of Senescence Associated-β-gal Activity

Senescence-associated (SA)-β-gal activity was quantitatively determined in control and knockdown HUVECs lysates 7 days after knockdown using a commercially available kit (Cell Biolabs). Samples were harvested and processed according to manufacturer’s instructions using 10µg of protein per assay. Total protein concentration was determined by the method of Bradford (BioRad). Activity was determined by fluorescence detection using a SpectraMax M5 plate reader (Molecular Devices). Results were expressed as fluorescence units/time/µg of protein.

### Pro-Inflammatory Phenotype Assay

The requirement of c-Myc for prevention of pro-inflammatory phenotype was evaluated by treatment of control and knockdown HUVECs 6 days after transduction with 0.1 ng/ml TNF-αfor 3 hours, and gene expression analysis of pro-inflammatory markers by RT-PCR.

### RNA Isolation and Gene Expression Profiling by Quantitative RT-PCR

Control and knockdown HUVECs lysates were collected and RNA extracted using RNeasy kit (Qiagen) according to manufacturer’s instruction. All samples were subjected to in-column DNAse treatment (Qiagen) prior to cDNA synthesis. First strand cDNA was prepared using High Capacity cDNA Reverse Transcriptase kit (Applied Biosciences, Life Technologies). Reverse- transcribed cDNA was used for Quantitative RT-PCR using pre-designed TaqMan Signature Arrays for Human Angiogenesis, Extracellular Matrix and Cell Adhesion Molecules, Cell Cycle Regulation (Applied Biosystems, Life technologies), and Endothelial Cell Biology RT^2^ PCR Array (SABiosciences, Qiagen). Samples tested in these arrays were collected from 3–5 independent experiments. Fold-changes in knockdown samples were calculated relative to NS-control by the ΔΔCt method using at least 2 endogenous controls for normalization. Network pathway analysis was performed using MetaCore software (Thomson Reuters), licensed to the University of Miami.

### Protein Expression Analysis by Western Blot and ELISA

Control and knockdown HUVECs lysates were prepared in RIPA buffer. Concentrated cell culture supernatants were collected 6 days after knockdown by incubating cultures the day before in half of the regularly used volume of EGM-2 media. We found this concentration approach to be sufficient for detection of cytokines in the supernatant. Protein was quantified in both RIPA lysates and culture supernatants using Bradford Assay (Bio-Rad). SDS-PAGE and Western blots were performed according to standard procedures using antibodies against c-Myc (Cell Signaling, #5605), p21 (Santa Cruz Biotechnologies, #SC-756), Cdc25A (Cell Signaling, #3652), Angptl4 (Abcam, #ab115798), Cxcl12 (Cell Signaling, #3740), Mdk (Abcam, #ab36038), Tgfb2, (Abcam, #ab113670), Tnfsf15 (Abcam, #ab21272), Vcam1 (Abcam,#ab134047), Actin (Sigma-Aldrich, #A2066). Commercially available ELISA kits were used to analyze the expression of Angptl4 (Abcam, #ab99974), Tgfb2 (Abcam, #ab100648) and Cxcl12 (Abcam, #ab23722) in culture supernatants and RIPA lysates. Densitometry Analysis of western blots was performed using Quantity One software (Bio-Rad).

### Statistical Analysis

Data obtained from all experiments were analyzed for significance using Student *t-*test comparing knockdown (KD) samples to control (NS), and One-Way ANOVA in TNF-α treatment experiments, with Microsoft Excel, Sigma-Plot and GraphPad Prism Software. p<0.05 was considered significant. All data are presented as means±standard error.

## Results

### c-Myc Knockdown in Endothelial Cells Reduces Proliferation and Morphogenesis

**Figure 1:**
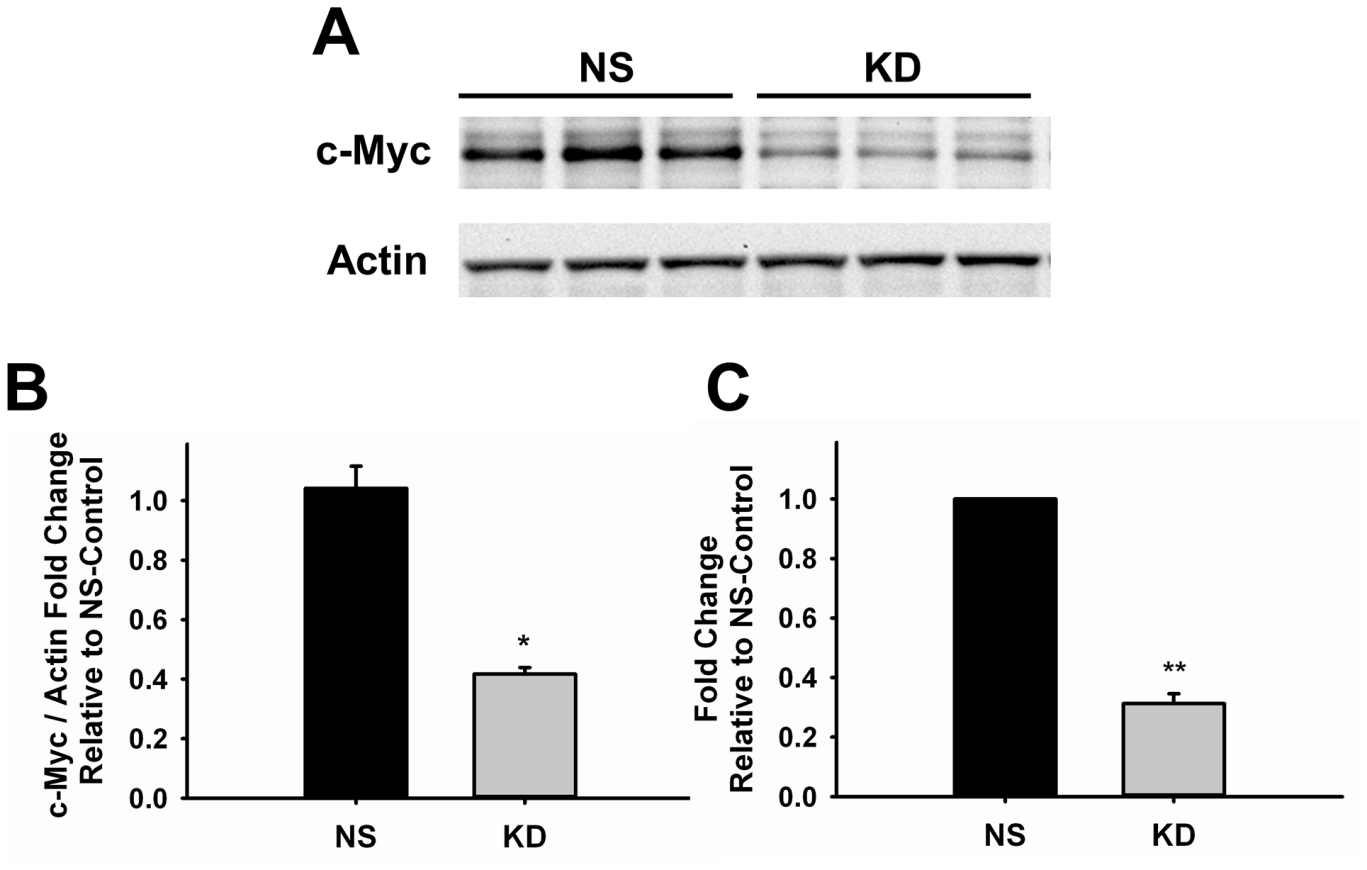
Knockdown of c-Myc in Endothelial Cells. **A.** Representative western blot image of control and knockdown samples of three independent experiments. Actin was used as loading control (n = 3) **B.** Quantification of c-Myc knockdown at protein level by densitometry analysis of western blot shown in A (n = 3). **C.** Quantitative RT-PCR analysis of control and knockdown samples (n = 6). Results are expressed as fold-change relative to NS-Control. Data were normalized to actin endogenous control gene (n = 4). *p<0.01, **p<0.001. NS, control; KD, knockdown.

The growth of new blood vessels depends on the proliferative and morphogenic potential of endothelial cells. In order to better understand how c-Myc regulates angiogenesis we transduced human umbilical vein endothelial cells (HUVECs) with lentiviral vectors expressing shRNA against c-Myc (KD) or non-silencing shRNA (NS) as control. The degree of knockdown achieved was 59.4±4.4% (p<0.01) and 68.8±3.4% (p<0.001) at protein and RNA, respectively (Figure 1).

**Figure 2:**
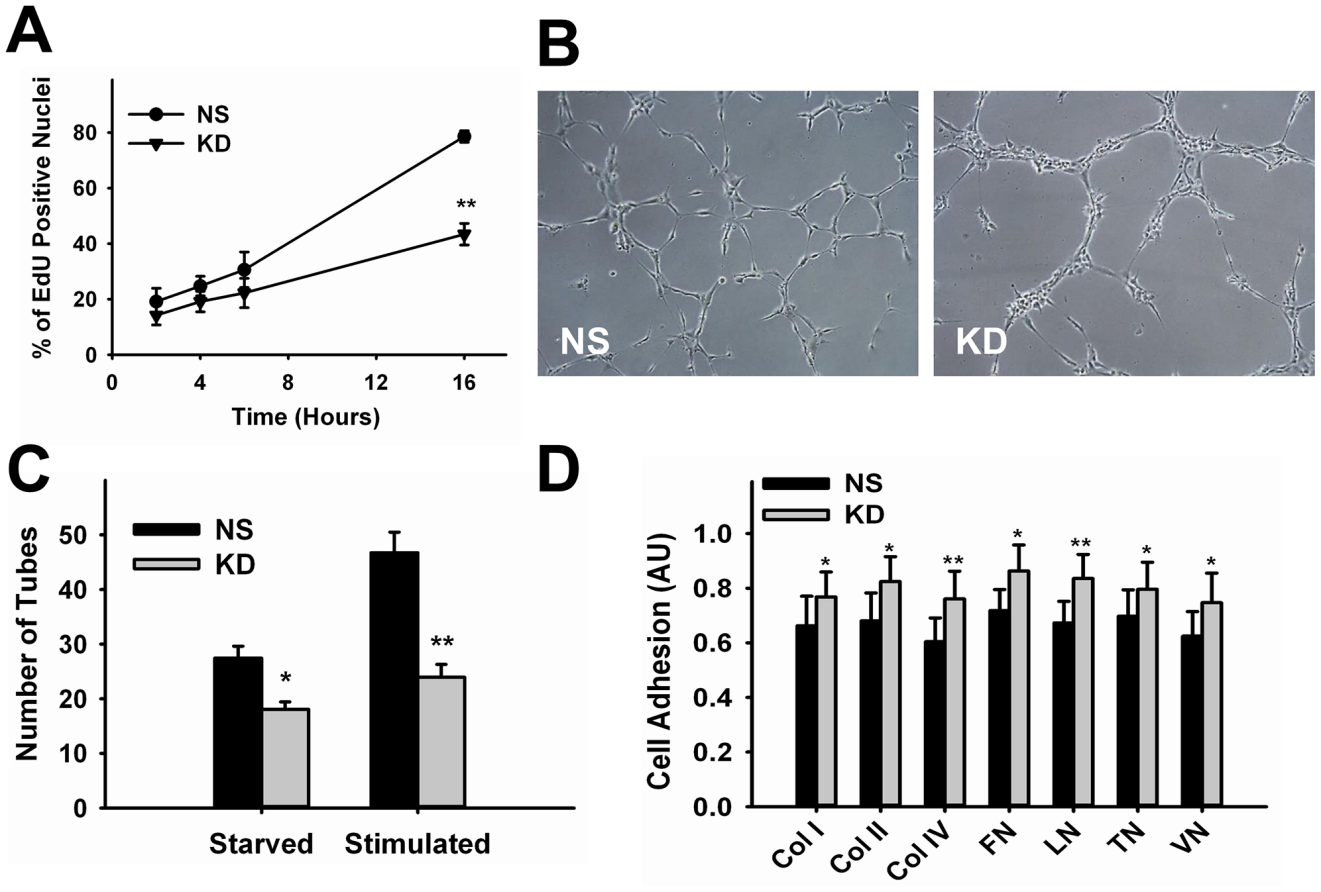
Effect of c-Myc Knockdown in Endothelial Cell Proliferation and Morphogenesis. **A.** Time-course analysis of DNA synthesis in control and knockdown cells from 2–16 hours (n = 3–7). **B.** Representative images of stimulated control and knockdown cells collected 5 hours after incubation in Matrigel. **C.** Quantitative analysis of tube formation (n = 3–6). **D.** Quantification of control and knockdown cell adhesion to extracellular-matrix proteins (n = 5). *p<0.05, **p<0.005. NS, control; KD, knockdown; Col, collagen; FN, fibronectin; LN, laminin; TN, tenascin; VN, vitronectin. Magnification = 10X.

We analyzed the effect of c-Myc knockdown on HUVECs proliferation by quantifying the rate of cell division through measurement of DNA synthesis. We observed that reduction in c-Myc expression significantly decreased proliferation. Importantly, no increase in cell death was observed along the period these cells were maintained in culture that could justify reduction in cell numbers. Analysis of DNA synthesis showed significant reduced rate of incorporation of the nucleoside analogue EdU in KD cells at 16 hours by 45.1±4.0% (p<0.00001) compared to NS-control (Figure 2A).

During early angiogenic stimulation, activated endothelial cells form tip and stalk cells that migrate towards growth factor gradients [20]. This morphogenic event in which endothelial cells form tubular structures can be replicated *in vitro* by culturing endothelial cells in basement membrane matrix (matrigel). To test if c-Myc loss affects endothelial cell tube formation potential, control and knockdown cells grown in complete media (stimulated) or starved overnight were plated on matrigel and analyzed after 5 hours for tube formation. Knockdown cells displayed a different distribution pattern on matrigel compared to control cells, mainly clustering attached to each other instead of dispersing along the matrix to form long tubular extensions similar to control cells (Figure 2B). Quantification showed that the number of tubes formed by KD cells was significantly reduced under starved (34.1±2.0%, p<0.05) and stimulated (48.7±3.2%, p<0.005) conditions (Figure 2C). The fact that reduction in tube formation was independent of the stimulation state of the cells suggests that the response to growth factors present in the matrigel may be compromised after c-Myc knockdown. Morphogenesis can be affected by cell-extracellular-matrix (ECM) interactions. In order to determine if c-Myc knockdown altered the adhesion of endothelial cells to ECM components, we performed an ECM-adhesion assay. We found that the adhesion of KD cells to different extracellular-matrix proteins was increased in average by 20.2±1.7% (Figure 2D). These results suggest that in addition to lack of response to growth factor stimulation, reduced morphogenesis caused by c-Myc knockdown also correlates with increased adhesion of endothelial cells to ECM, perhaps impairing proper migration and tip formation.

### c-Myc Knockdown in Endothelial Cells Triggers Senescence

**Figure 3:**
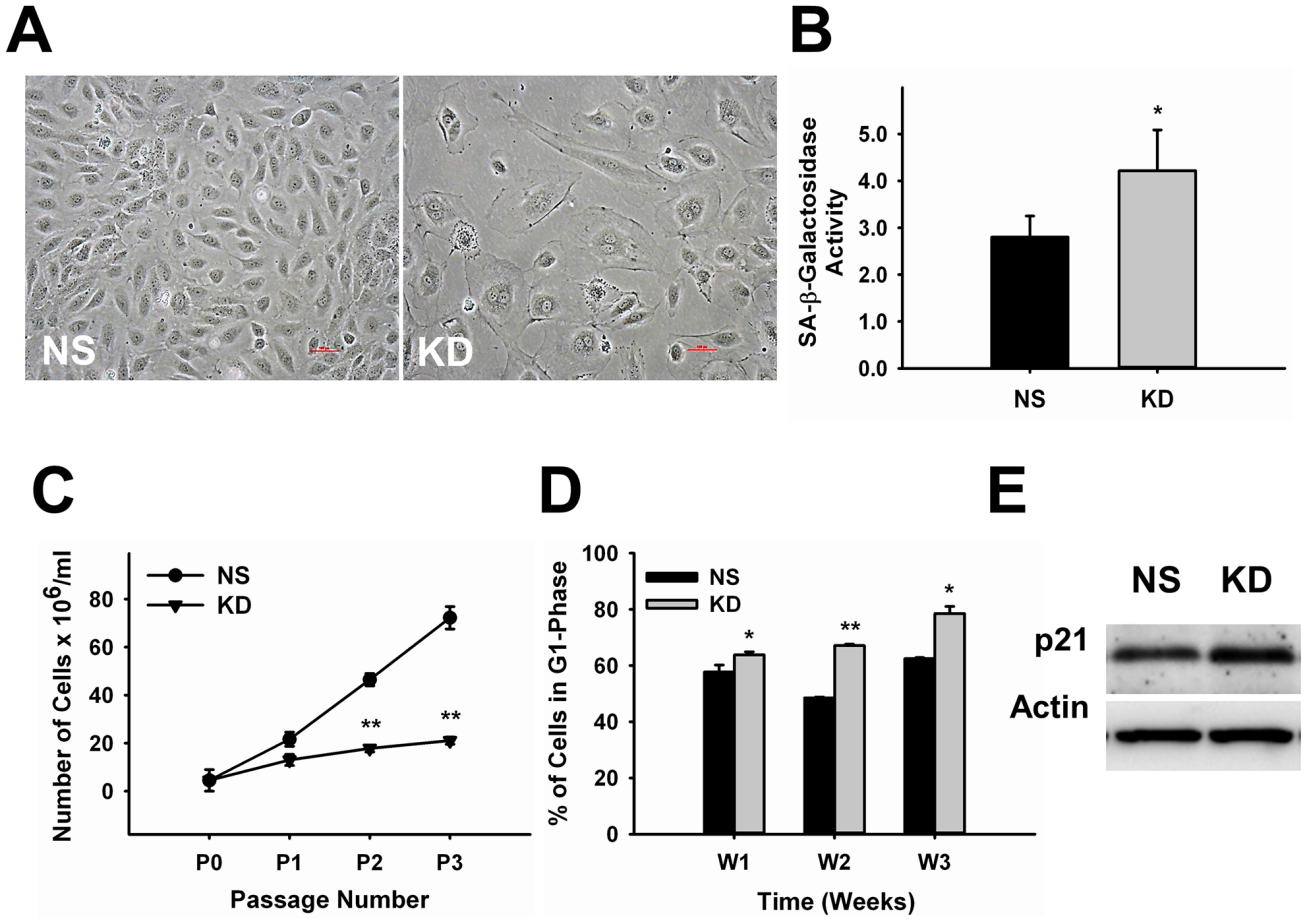
c-Myc Knockdown in Endothelial Cells Leads to Senescence. **A.** Representative images showing morphological changes associated with senescence in c-Myc deficient endothelial cells compared to NS-control. **B.** Quantitative analysis of senescence-associated-β-galactosidase activity in control and knockdown endothelial cells (n = 8). **C.** Determination of population doubling along passage (n = 8). **D.** Time-course cell cycle analysis of control and knockdown endothelial cells showing accumulation in G1-phase (n = 3–7). **E.** Representative western blot image of control and knockdown cells showing upregulation of the cell cycle inhibitor p21, 3–4 days after knockdown. Actin was used as loading control (n = 3). *p<0.05, **p<0.005. NS, control; KD, knockdown. Magnification = 20X.

Defective proliferation and morphogenesis after c-Myc knockdown in endothelial cells was accompanied by significant morphological changes typically found in senescent cells. Senescence is accompanied by morphological changes, upregulation of cell cycle inhibitors and downregulation of activators leading to cell cycle arrest at G1-phase, and increased levels of senescence-associated (SA)-β-galactosidase activity. Consistent with this, we found that c-Myc-deficient endothelial cells presented a large-flat morphological appearance and binucleation (Figure 3A). SA-β-galactosidase activity was significantly increased 47.2±8.7% (p<0.05) in KD cells relative to NS-control, 7 days after knockdown (Figure 3B). Differently from quiescence, during senescence cells undergo irreversible cell cycle arrest and cannot reenter the cell cycle. Quantification of population doubling, another parameter used to determine senescence, indicated that the number of KD cells was stabilized with passage and did not increase with time compared to NS-control cells (Figure 3C). Cell cycle analysis over a period of 3 weeks showed a time-dependent accumulation of c-Myc knockdown cells in G1-phase relative to control (Figure 3D). The first signs of delay in cell cycle progression were observed one week after c-Myc knockdown; 63.8±1.1% KD cells were distributed in G1-phase compared to 57.7±2.5% NS-Control cells (∼10% increase, p<0.05). By the end of three weeks, the number of KD cells in G1-phase was increased ∼24% (78.5±2.5 KD vs. 62.5±0.4 NS-control, p<0.05). c-Myc is known to control the expression of different cell cycle proteins and to induce senescence through mechanisms that involve upregulation of the cell cycle inhibitor p21 [21], [22]. Accordingly, western blot analysis showed that p21 expression was increased approximately 29.2±3% (p<0.003) in KD cells (Figure 3E). Altogether, these results suggest that reduction in c-Myc expression levels in endothelial cells trigger senescence.

### Gene Expression Analysis of c-Myc-deficient Endothelial Cells

In order to determine possible downstream targets of c-Myc that control endothelial function, we performed gene expression analysis by quantitative RT-PCR using commercially available PCR-arrays for angiogenesis/endothelial cell biology, extracellular-matrix (ECM)/adhesion molecules and cell cycle regulation genes. A complete list of significant gene expression changes 7 days after knockdown is shown in Table 1. The results confirm increased expression of several growth factors, cytokines, extracellular-matrix proteins and adhesion molecules in knockdown cells (Table 1) reflecting an activated endothelial state. A number of these, including cytokines Angptl4 (4.66-fold), Mdk (2.82-fold), Tgfb2 (4.56-fold), Tnfsf15 (4.61-fold); chemokines Cxcl2 (3.83-fold) and Cxcl12 (6.32-fold), and cell adhesion molecules Icam1 (4.07-fold) and Vcam1 (4.27-fold), are pro-inflammatory molecules. The expression of regulators of vascular tone was also altered. Angiotensin converting enzyme (Ace) was downregulated (1.7-fold); while endothelin-1 (Edn1) and endothelin receptor A (EdnrA) were both upregulated (2.01- and 4.19-fold, respectively). As loss of c-Myc had a significant impact in endothelial cell proliferation, we also analyzed control and knockdown cells for the expression of cell cycle-related genes. Cell cycle inhibitor genes Cdkn1a (p21) and Cdkn2d (p19) were significantly upregulated 1.93- and 1.94-fold in KD cells, relative to control cells, while the cell cycle activators Ccnb1 (Cyclin B1) and Cdc25A (Cell Division Cycle Homolog 25A) were reduced 1.84- and 1.80-fold, respectively. This gene expression signature is consistent with a typical activated pro-inflammatory-secretory phenotype triggered during cellular senescence.

**Table 1:** Gene Expression Analysis of HUVECs 7 Days after c-Myc Knockdown.

Gene	FoldChange	p-value	Gene	FoldChange	p-value
CXCL12	6.32	0.0089	PDGFB	2.00	0.0003
ANGPTL4	4.66	0.0166	IL8	1.98	0.0186
TNFSF15	4.61	0.0001	COL6A2	1.94	0.0338
TGFB2	4.56	0.0050	CDKN2D	1.94	0.0033
VCAM1	4.27	0.0067	CDKN1A	1.93	0.0062
KIT	4.23	0.0001	CDH5	1.91	0.0013
EDNRA	4.19	0.0100	FGF1	1.89	0.0316
ICAM1	4.07	0.0101	HSPG2	1.84	0.0235
CXCL2	3.83	0.0153	CTGF	1.83	0.0071
TYMP	3.34	0.0041	COL4A2	1.83	0.0044
MDK	2.82	0.0265	COL5A1	1.83	0.0192
HDAC9	2.89	0.0046	ITGA3	1.81	0.0148
FN1	2.61	0.0113	COL4A1	1.79	0.0002
COL8A1	2.50	0.0012	TIMP3	1.72	0.0508
ITGB2	2.39	0.0064	MMP10	1.65	0.0294
ITGAV	2.32	0.0008	TIMP2	1.63	0.0262
ECM1	2.24	0.0041	GRN	1.64	0.0112
PECAM1	2.15	0.0119	COL12A1	1.62	0.0405
MMP2	2.14	0.0092	FLT1	1.62	0.0255
ITGA1	2.11	0.0051	ITGA5	1.58	0.0505
TGFBI	2.10	0.0096	THBS1	1.56	0.0233
TEK	2.10	0.0067	MMP1	0.60	0.0163
EDIL3	2.05	0.0005	ACE	0.61	0.0200
KDR	2.03	0.0031	CDC25A	0.56	0.0350
EDN1	2.01	0.0006	CCNB1	0.54	0.0282

### Induction of Pro-inflammatory Phenotype in c-Myc Deficient Endothelial Cells

**Figure 4:**
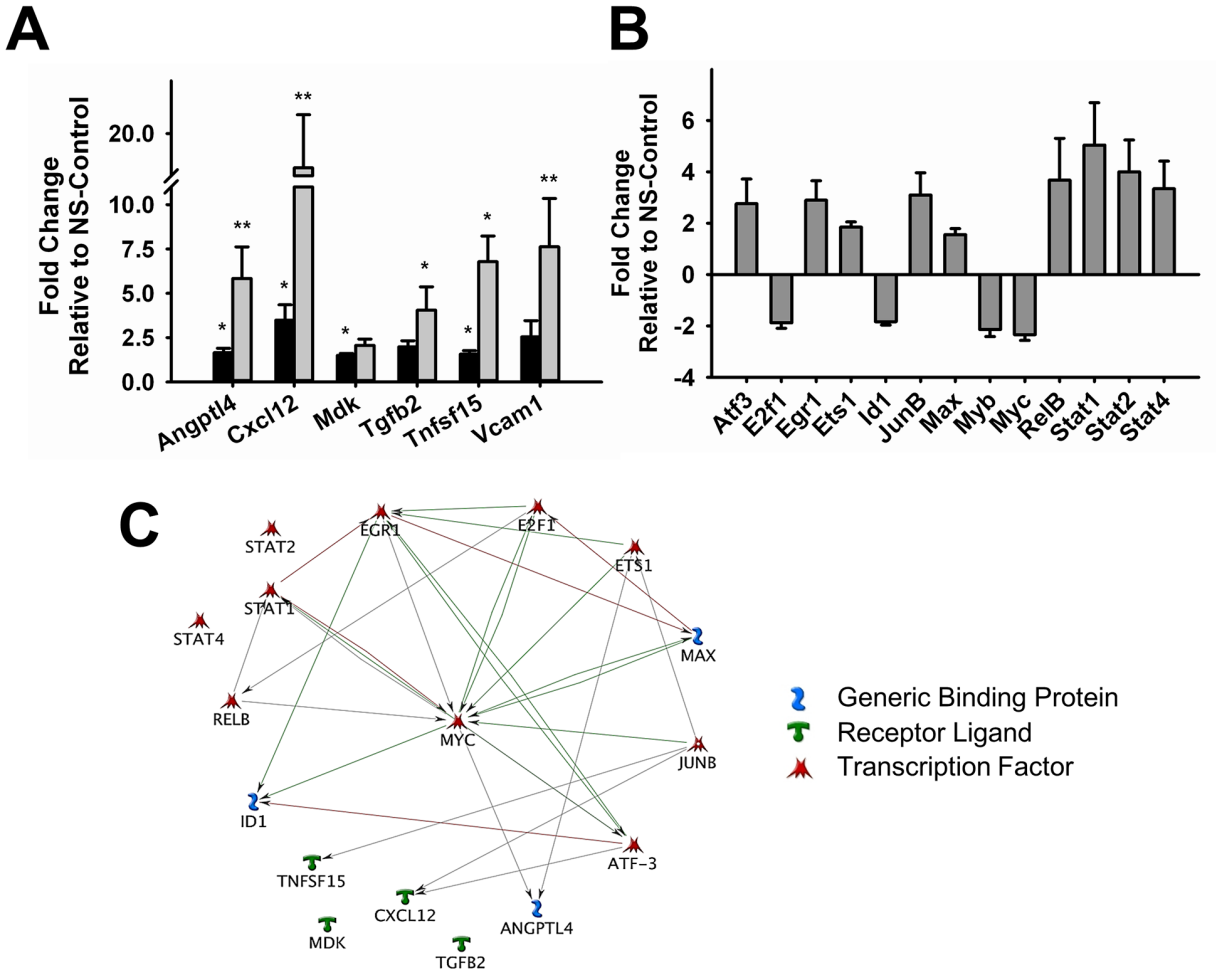
Gene Expression Analysis of Inflammatory Mediators and Transcription Factors after c-Myc knockdown. **A.** Time-dependent changes in the expression of pro-inflammatory genes Angptl4, Cxcl12, Mdk, Tgfb2, Tnfsf15 and Vcam1 in endothelial cells three (black bars) and six (gray bars) days after c-Myc knockdown. **B.** Transcription factor gene expression profiling 6 days after c-Myc knockdown. Results are expressed as fold-change relative to NS-Control. Gene expression data were normalized to at least two endogenous control genes. **C.** Network pathway analysis of inflammatory mediators and transcription factors induced by c-Myc knockdown. (n = 4–5) (*p<0.05, **p<0.005).

**Figure 5:**
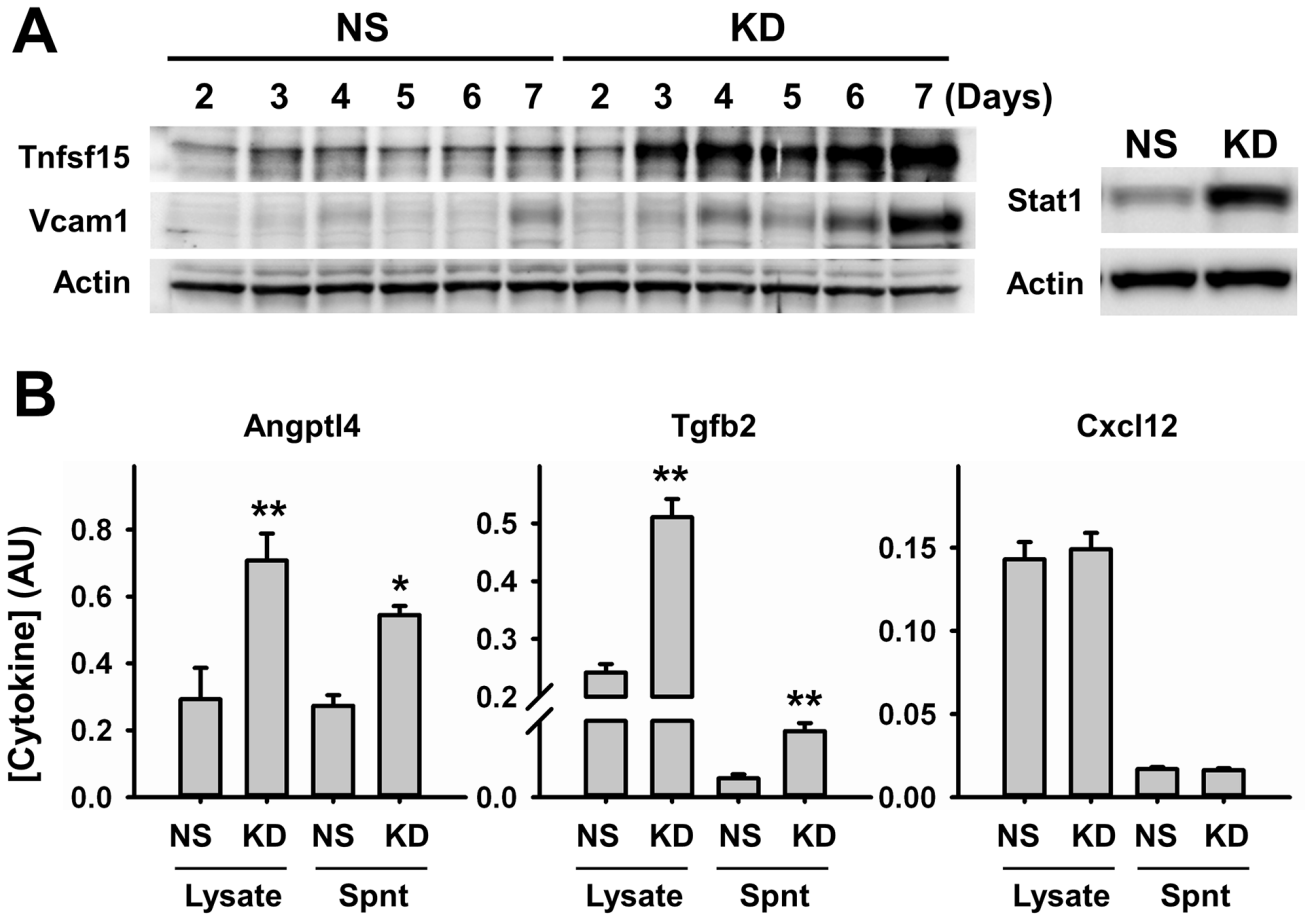
Protein Expression Analysis of Pro-Inflammatory Markers. **A.** Representative western blot image showing time-dependent changes in the expression of Tnfsf15, Vcam1, and Stat1 after c-Myc knockdown. Expression of Stat1 was analyzed 6 days after knockdown. Actin was used as loading control (n = 4). **B.** Expression analysis of Angptl4, Tgfb2 and Cxcl12 in cell lysates and concentrated supernatants (Spnt) by ELISA 6 days after knockdown (n = 4). NS, control; KD, knockdown. *p<0.05, **p<0.005.

One of the most detrimental consequences of senescence is the development of chronic low-level inflammation, which contributes to several pathological conditions [23], [24]. Therefore, we decided to investigate potential mechanisms activated by c-Myc knockdown that may trigger this response. We performed time-course studies to follow changes in the expression of target inflammatory mediators identified in our profiling studies, Angptl4, Cxcl12, Mdk, Tgfb2 and Tnfsf15. We observed that RNA levels of most of these targets are significantly upregulated early during knockdown (day 3). However expression increases with time and peaks at a later time point (day 6) during a period of 1 week (Figure 4A). Significant knockdown of c-Myc is evident 48–72 hours after transduction and remains constant during the course of our studies (data not shown), suggesting that other transcription factors may be involved in the delayed peak of inflammatory cytokine expression. Gene expression analysis at this time point revealed that transcription factors known to be involved in growth regulation (E2f1, Id1 and Myb) were downregulated, while those involved in inflammatory responses (RelB, Stat1, Stat2 and Stat4) were upregulated (Figure 4B). Pathway analysis showed that most of these transcription factor genes have been previously reported to interact with c-Myc and with each other. However, except for Angptl4, none of the target cytokines we describe in our study have been previously shown to be a downstream target of c-Myc. Interestingly, no interactions were observed with Mdk and Tgfb2 for any of the transcription factors, including c-Myc (Figure 4C). We performed western blot analysis and ELISA, to confirm if the transcriptional changes we observed were also reflected at protein level. Our results confirm that c-Myc knockdown also significantly increased the expression of Tnfsf15, Angptl4, Tgfb2 and Vcam1 proteins (Figure 5). Although Cxcl12 transcripts were considerably upregulated compared to all others, we were not able to detect any significant changes in Cxcl12 protein expression using different methods or samples (Figure 5B). Among the transcription factors that we found upregulated in our arrays, Stat1 showed the highest induction in expression. This increase was also confirmed at protein level (Figure 5A).

### c-Myc is Downregulated in Replicative- and Stress-induced Senescence

**Figure 6:**
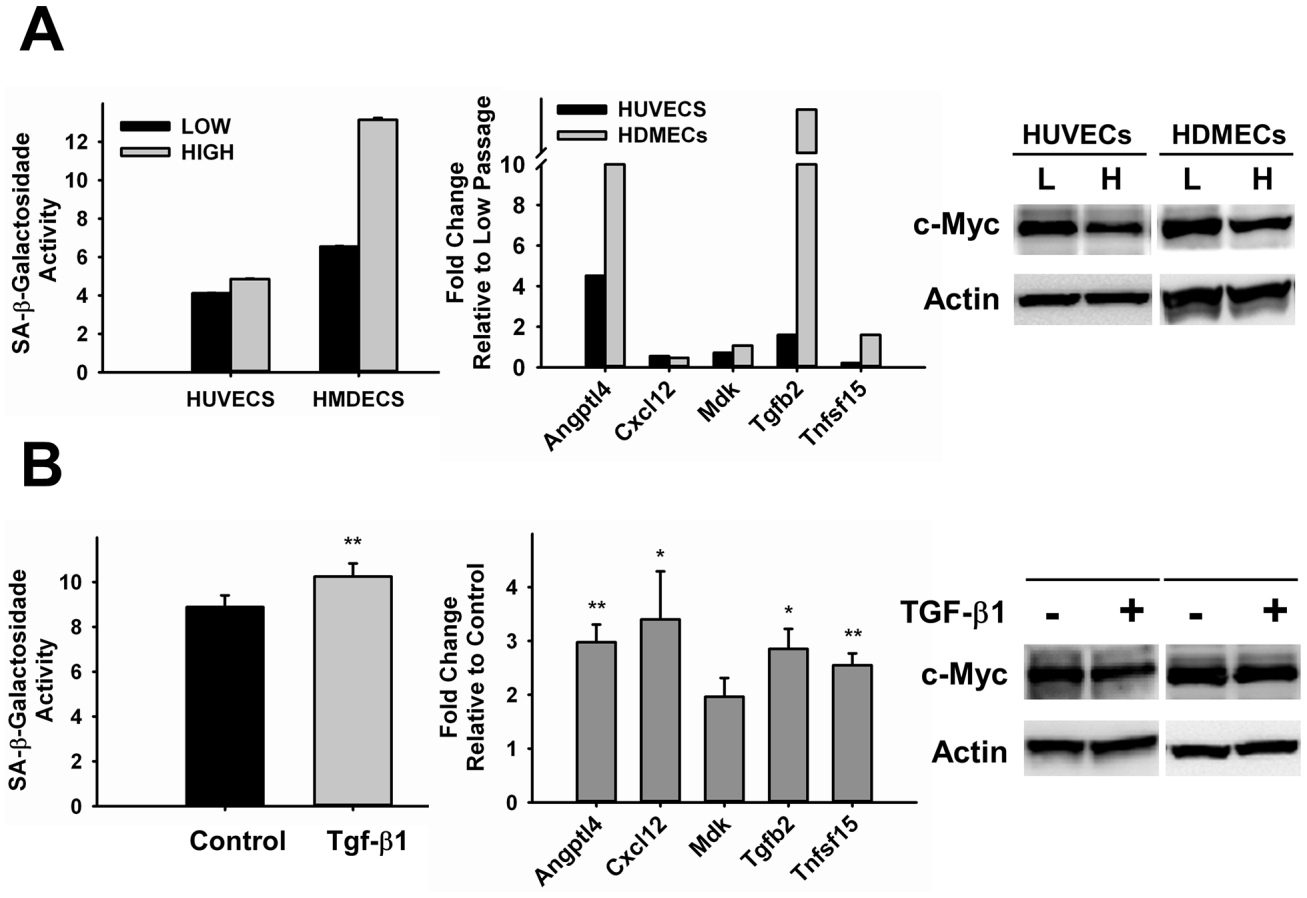
Analysis of c-Myc Expression in Endothelial Cells Undergoing Replicative- or Stress-Induced Senescence. **A.** HUVECs and HMDECs at low (P6) and high (P12–11) passages. **B.** HUVECs treated with TGF-β1 for three days (n = 4–6). All samples were analyzed for SA-β-galactosidase activity (right panel), expression of pro-inflammatory markers genes (middle panel) and c-Myc expression by Western Blot (right panel). Actin was used as loading control. L = Low, H = High. *p<0.05, **p<0.005.

In order to demonstrate that c-Myc expression is normally downregulated in endothelial cells undergoing senescence, we performed studies using conditions known to lead to senescence. We first investigated the expression of c-Myc in replicative senescence, a phenomenon that occurs after cells undergo a certain number of cell divisions [25], [26]. The length of time or passages required for induction of senescence is cell-specific. We compared HUVECs and human dermal microvascular endothelial cells (HDMECs) at low (P6) and higher (P12 and P11, respectively) passages. Morphological senescence-related changes were more evident in high passage cultures of HDMECs than in HUVECs (data not shown). The level of SA-β-galactosidase activity was increased 18% in HUVECs and 100% in HMDECs (Figure 6A, left panel). As expected, cellular aging leads to upregulation of pro-inflammatory markers (Figure 6A, middle panel). We focused on expression changes of the cytokines targeted by c-Myc knockdown in our study. Interestingly, the effect was cytokine specific. Most significant changes were observed in Angptl4 and Tgfb2 expression, and consistent with the level of SA-β-galactosidase activity in each cell line. Downregulation of c-Myc in high passage cells was observed in both cases (Figure 6A, right panel). In addition to replicative senescence, we investigated the effect of inflammatory stress-induced senescence in c-Myc expression in endothelial cells. Members of the TGF-β family have been previously shown to be upregulated with aging in humans and animal models and to be pro-senescence [27], [28]. HUVECs were allowed to reach confluence to induce a quiescence state and treated with TGF-β1 diluted in low-serum condition. Three days after treatment, TGF-β1 significantly induced SA-β-galactosidade activity by 15.6±2.8% (p<0.001) (Figure 6B, left panel). These results coincided with reduction in c-Myc expression by approximately 22.5±6.7% (p<0.04) (Figure 6B, right panel). Regarding development of pro-inflammatory phenotype, we found all cytokines investigated to be significantly upregulated in TGF-β1-induced senescence. Mdk was the only exception, but still showed a trend of induction, although not significant (Figure 6B, middle panel). Altogether, these results confirm that c-Myc expression is reduced when endothelial cells undergo physiological senescence and that this effect coincides with the development of pro-inflammatory phenotype.

### c-Myc Knockdown Increases Pro-inflammatory Response

**Figure 7:**
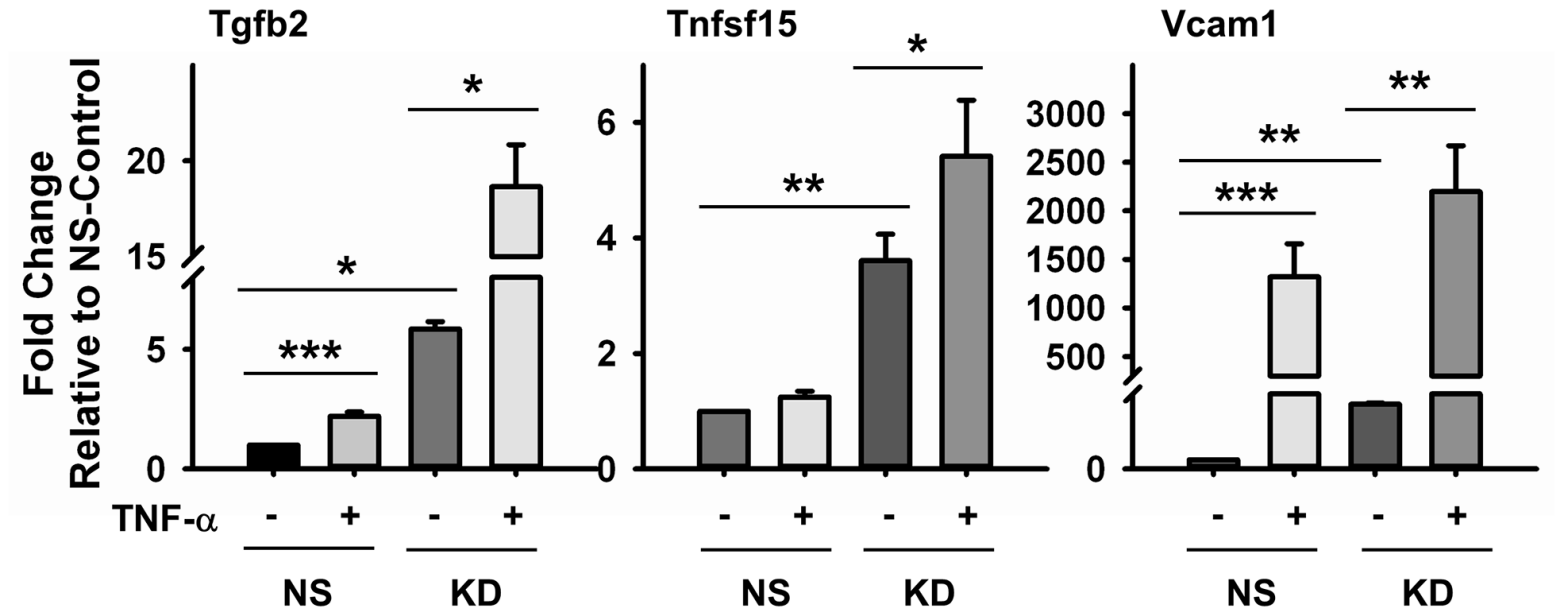
Effect of TNF-α in c-Myc deficient HUVECs Pro-inflammatory Response. Control and Knockdown HUVECs were treated with TNF-α for three hours and analyzed for expression of pro-inflammatory genes by RT-PCR. Results are expressed as fold-change relative to NS-Control. Data were normalized to at least two endogenous control genes. *p<0.05, **p<0.005. ***p<0.0005, (n = 3).

Our results show that knockdown of c-Myc in endothelial cells leads to development of a pro-inflammatory response. To determine the requirement of c-Myc to prevent or reduce inflammation, we treated control and knockdown HUVECs with TNF-α, a cytokine known for its pro-inflammatory effects. Gene expression analysis showed that among the inflammatory markers we found to be upregulated by c-Myc knockdown, TNF-α treatment of NS-Control cells significantly induced the expression of Tgfb2 and Vcam1. In addition, there was a mild induction in Tnfsf15 expression, which was not significant (Figure 7). c-Myc knockdown cells treated with TNF-α developed a significantly enhanced pro-inflammatory response relative to NS-Control (untreated and treated) and untreated knockdown cells (Figure 7). These results support our findings that reduced c-Myc expression in endothelial cells makes them more prone to develop a pro-inflammatory phenotype.

## Discussion

In the present study we provide the first evidence that reduction in c-Myc expression in endothelial cells triggers a pro-inflammatory senescent phenotype, features typically observed during vascular aging and pathologies associated with endothelial dysfunction. Previous studies on c-Myc in the vasculature have mostly been focused on smooth muscle cell proliferation in vascular injury models [6], [9]. More recently, we and others have shown that c-Myc plays a role during vascular development *in vivo* [10]–[13]. However, the molecular mechanisms involved were not fully clarified. Vascular defects were evident during angiogenesis and were related to contradictory phenotypic alterations in morphogenesis, endothelial cell proliferation, survival and vascular permeability. One common feature suggested by these studies is that c-Myc is required for blood vessel maturation. Throughout this stage, endothelial cells must actively proliferate to expand and shape the vascular tree [29].

To obtain new insights on potential mechanisms related to c-Myc role in the vasculature, we performed a series of *in vitro* studies and gene expression profiling in endothelial cells expressing reduced c-Myc levels. We found that c-Myc knockdown significantly decreased endothelial cell proliferation, what could account for reduced morphogenesis observed in previous studies during vascular development. c-Myc drives proliferation of many different cell types, though its role in endothelial cell proliferation has been controversial [10]–[13]. In support of our current observations, we have previously shown in *Xenopus* embryos that c-Myc knockdown or overexpression caused a decrease or increase in the number of endothelial cells, respectively [11]. Contrarily, He et al reported in a mouse model that endothelial-specific deletion of c-Myc *in vivo* did not show any significant effects on proliferation rates [12]. However, significant increase in cell death at the embryonic stage investigated was reported, which could explain the observed lack of effect on proliferation. Moreover, the effects we observed on endothelial proliferation after c-Myc knockdown were associated with development of senescence, as confirmed by several parameters, including population doubling assays and defective morphogenesis. Therefore, the lack of proliferation and reduced morphogenesis in this mouse model are likely due to endothelial senescence, what has not been addressed. In general, overexpression of c-Myc has been related to apoptosis and senescence as a defense mechanism to control deregulated cell growth [30], [31]. However, similarly to what we observed, some reports have described that development of senescence in different cell types could also be associated with reduction in c-Myc expression, through mechanisms that involve upregulation of cell cycle inhibitors such as p15, p16 and p21 [32]–[34]. The effects we observed after c-Myc knockdown were specifically associated with downregulation of Cdc25A and Cyclin B1 and upregulation of p19 and p21. The expression of c-Myc has been previously shown to be required for transcriptional repression of p21 and p15 [22], [35]. Therefore, reduction in c-Myc levels in endothelial cells may lift repression of these cell cycle inhibitors triggering cell cycle arrest. The choice between reversible or irreversible cell cycle arrest (senescence) may depend on the activity of other cell cycle regulators. The cyclin dependent kinase-2 (Cdk2) is known to participate in c-Myc growth control [21] and its activity is regulated during G1/S-phase transition by Cdc25A [36]. Downregulation of Cdc25A expression in endothelial cells as we observed after c-Myc knockdown suggests that, in addition to upregulation of cell cycle inhibitors, reduction in Cdk2 activity may contribute to senescent fate.

Senescent endothelial cells are metabolically active, secreting growth factors and chemokines, and expressing high levels of adhesion molecules that enhance recruitment and attachment of inflammatory cells [17]. These alterations gradually compromise endothelial function by causing phenotypic and hemodynamic changes, increasing the risk of cardiovascular disease (CVD) [17]. Therefore, it is crucial to understand and determine possible mechanisms and targets that trigger endothelial senescence and endothelial dysfunction in order to improve early detection and prognosis of CVD. The major novel finding of our work relates to development of a pro-inflammatory phenotype by c-Myc knockdown in endothelial cells. Our gene profiling study showed that c-Myc regulated the expression of several pro- and anti-angiogenic growth factors, as well as pro-inflammatory cytokines and adhesion molecules involved in recruitment and attachment of inflammatory cells to endothelial cells. Among novel potential downstream targets of c-Myc, significant changes in the expression of Angptl4, Mdk, Cxcl12, Tgfb2 and Tnfsf15 were observed. Angptl4 is upregulated during acute inflammation in different tissues where it may play a protective role [37]. In the endothelium, there are controversial reports that Angptl4 has pro- and anti-angiogenic properties [38], [39]. Many studies have focused on the role of Angptl4 in the control of vascular permeability through interaction with integrins and adhesion molecules present in endothelial cells [40]. Mdk is a growth factor associated with acute and chronic inflammatory processes, which promotes the expression of chemokines, leukocyte migration, and suppression of regulatory T-cells expansion [41]. Mdk is induced in injured endothelium *in vivo*, and has been associated with neointima formation [42]. In addition to its role in inflammatory processes, like Angptl4, Mdk also plays contradictory roles in angiogenesis [43], [44]. Cxcl12 (Sdf-1) plays a central role in regulating angiogenesis by promoting endothelial cell chemotaxis and mitogenesis [45]. In addition, during vascular injury, Cxcl12 can also contribute to inflammation and neointimal hyperplasia by recruitment of specific lineage−/PDGFβR^+^/Sca1^+^ progenitor cells that give rise to smooth muscle cells [46], and by orchestrating chemoattraction and transendothelial migration of leukocytes [47]. In vascular pathologies, TGF-β is well known for its regulatory role of inflammatory responses and tissue remodeling [48]. Upregulation of TGF-β in response to vascular injury is thought to be crucial for development of vascular inflammation [49]. Furthermore, TGF-β is a potent chemoattractant and stimulates the expression of inflammatory cytokines and adhesion molecules involved in cell-cell interactions [50]. Tnfsf15, also known as vascular endothelial growth inhibitor (VEGI) is a potent anti-angiogenic factor, promoting inhibition of endothelial cell proliferation and morphogenesis [51], [52]. Overexpression of Tnfsf15 has been previously shown to induce senescence of both human endothelial and vascular progenitor cells [53]. Interestingly, treatment of bovine aortic endothelial cells with Tnfsf15 prior to growth stimulation inhibited phospho-Rb hyperphosphorylation and induction of c-Myc [54], suggesting a regulatory loop between c-Myc and Tnfsf15 in the control of endothelial cell growth and senescence response. In addition to its role in the control of endothelial growth, Tnfsf15 has been shown to participate in inflammatory diseases [55]–[59] by directly controlling inflammatory response [60]–[63]. We found that c-Myc induced changes in Cxcl12 expression occurred mainly at RNA level. Similar results have been previously described in senescence-associated secretory phenotype in human fibroblasts and in inflammatory events, indicating tight regulation of inflammatory responses at post-transcriptional level, which is an important mechanism to protect tissues from injury [64], [65]. Our results suggest that this control may be cytokine specific. Induction of pro-inflammatory cytokines upon c-Myc knockdown is an early event, but the peak of expression occurs a few days later. Although reduction in c-Myc levels may relieve transcriptional repression of inflammatory genes, this time-dependent accumulation suggests that other factors downstream of c-Myc are involved in this regulation. Our results show that the expression of several transcription factors is induced at the peak of expression of pro-inflammatory cytokines suggesting activation of multiple pathways after c-Myc knockdown that may interact with each other to control this response. It is possible that the cytokines initially induced by c-Myc knockdown trigger this second-wave of inflammatory marker expression. Alternatively, c-Myc knockdown may trigger mechanisms involved in the control of RNA stability. Independent of the pathways involved, reduced c-Myc expression likely pre-prime the endothelium to inflammation, which can be triggered by external stress factors. Our TNF-α experiments showing that c-Myc deficient endothelial cells develop an enhanced inflammatory response compared to control cells support this idea.

Endothelial cells play an essential role in maintaining vascular homeostasis by regulating immuno-inflammatory responses, coagulation, neoangiogenesis after injury and alterations in blood flow [14]. Our findings support an essential physiological role for c-Myc in vascular homeostasis positioning this transcription factor as a central regulator of pro-inflammatory phenotype and endothelial dysfunction. We showed that c-Myc dowregulation is a regular trait of cells undergoing replicative and stress-induced senescence, and coincide with development of pro-inflammatory phenotype. As a master regulator of the genome, c-Myc is a perfect candidate to fine tune this complex balanced system. c-Myc is likely to play an important role during stress response in the endothelium. Transient changes in c-Myc expression were reported in endothelial cells during pathological levels of cyclic strain [66] and shear stress [67]. Guney et al have shown that low levels of oxidative stress could lead to down regulation of c-Myc in endothelial cells contributing to senescence [33]. Because of the extensive repertoire of key regulatory downstream targets, even transient changes in c-Myc expression could lead to cumulative detrimental effects in endothelial cells. The significance of our studies can be extended to pathological conditions related to endothelial dysfunction, such as diabetes-associated angiopathies. Both Mdk and TGF-β have been implicated in diabetic nephropathy in animal models and humans [68]–[70]. Angptl4 overproduction is associated with development of nephrotic syndrome [71], which can occur in subsets of diabetic patients [72]. Moreover, our findings are relevant to disease models of chronic inflammation and related increased risk of cancer [73], [74], possibly due to the pro-inflammatory/tumorigenic effect of the secretory senescent phenotype found in these conditions [24]. A close link between inflammation and cancer has been previously reported [75], [76]. Angptl4, Cxcl12 and Mdk, which are upregulated in our model, have all been implicated in cancer development and progression [77]–[79].

Future in vivo studies will confirm a potential role for c-Myc in vascular senescence and dysfunction. Further time-course studies are required to determine the precise cascade of events regulated by c-Myc that control the pro-inflammatory response, promoting inflammation and endothelial dysfunction. Identification of the stress signals that regulate c-Myc expression in endothelial cells may provide new information on the pathways that control endothelial dysfunction and senescence thereby revealing new therapeutic targets for prevention of cardiovascular disease.
